# Anterior segment optical coherence tomography and retained vegetal intraocular foreign body masquerading as chronic anterior uveitis

**DOI:** 10.1186/s12348-017-0130-7

**Published:** 2017-05-23

**Authors:** Anis Mahmoud, Riadh Messaoud, Fatma Abid, Imen Ksiaa, Melek Bouzayene, Moncef Khairallah

**Affiliations:** 1 Department of Ophthalmology, Taher Sfar University Hospital, Faculty of Medicine, University of Monastir, Monastir, Tunisia; 2 Department of Ophthalmology, Fattouma Bourguiba University Hospital, Faculty of Medicine, University of Monastir, 5019 Monastir, Tunisia

**Keywords:** Vegetal intraocular foreign body, Anterior segment optical coherence tomography, Masquerade syndrome

## Abstract

**Background:**

The purpose of this single case report was to report the use of anterior segment optical coherence tomography for the diagnosis and management of a retained vegetal intraocular foreign body.

**Results:**

A 23-year-old otherwise healthy male presented with a progressive vision loss in the right eye (RE). He reported a mild ocular trauma with a tree leaf 1 year ago followed by recurrent episodes of redness and pain in the RE that partially resolved after a self-medication with topical steroids. Visual acuity of the RE was limited to light perception. Slit-lamp examination of the RE showed an iris granuloma with overlying exudate and associated anterior chamber inflammatory reaction. Film X-rays, contact B-scan ultrasonography, and CT scan showed no abnormalities. Anterior segment optical coherence tomography revealed an enclaved iris foreign body. The foreign body was removed after a short course of local antibio-corticosteroid therapy. This was followed 2 months later by cataract surgery with intraocular lens implantation, with subsequent improvement of visual acuity to 20/40.

**Conclusions:**

A missed intraocular foreign body can lead to sight-threatening complications. Anterior segment optical coherence tomography may be useful for detecting non-clinically evident intraocular foreign body involving the anterior segment masquerading as chronic anterior uveitis.

## Findings

### Introduction

Intraocular foreign bodies (IOFBs) account for 18 to 41% of all open-globe injuries [1]. Anterior segment of the globe is the second most frequent location after the posterior one [1]. Missed IOFB may present with atypical clinical features that may lead to delay in correct diagnosis and in appropriate management [2, 3].

We herein report a case of small missed retained vegetal intracameral foreign body masquerading as chronic intraocular inflammation that could be detected only by anterior segment optical coherence tomography (AS-OCT).

### Case report

**Fig. 1:**
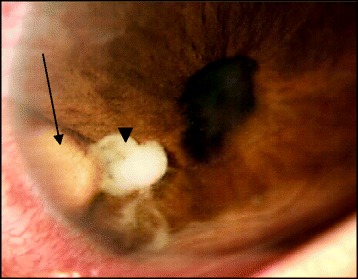
Slit-lamp photograph showing an iris granuloma (*arrow*) with adjacent exudate (*arrowhead*) and irregular pupil with posterior synechiae

A 23-year-old otherwise healthy male presented with a progressive visual loss in the right eye (RE). He reported a mild ocular trauma with a tree leaf 1 year ago followed by recurrent episodes of redness and pain in the RE that partially resolved after a self-medication with topical steroids. On ocular examination, his visual acuity was of light perception in the RE and 20/20 in the left eye (LE). Slit-lamp examination of the RE showed a normal cornea, fine keratic precipitates, 2+ cells in the anterior chamber, and an iris granuloma with adjacent inflammatory exudate. The anterior chamber depth was irregular with irido-capsular lens adhesions, and a mature cataract was noted (Fig. 1). Intraocular pressure was 11 mmHg. Posterior segment examination was prevented by the poor pupil dilation and the presence of mature cataract, and no entry wound was detected. Results of anterior segment and fundus examination of the LE showed no abnormalities. B-ultrasonography was performed and revealed mild vitritis.

Results of chest X-ray, blood cell count, C reactive protein, PPD, and syphilis serology were normal or negative. A plain orbital X-ray, ocular ultrasonography, and orbital CT scan showed no evident IOFB. UBM was not available.

**Fig. 2:**
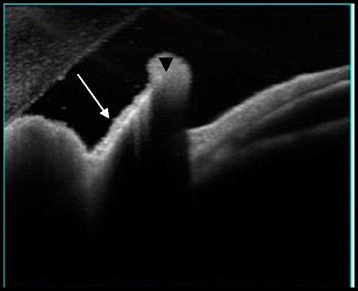
High resolution AS-OCT showing a linear highly reflective structure with marked posterior shadowing suggesting a foreign body (*arrow*) retained in the iris. Note the presence of adjacent moderate hyper-reflectivity (*arrowhead*) corresponding to the inflammatory exudate seen clinically

**Fig. 3:**
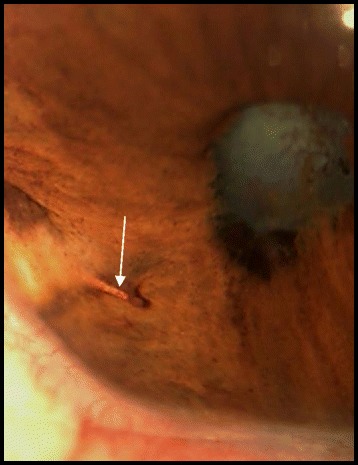
On slit-lamp photography after 2 weeks of topical corticosteroids, the IOFB became evident after decrease in the granuloma size and resolution of associated inflammatory exudate (*arrow*)

**Fig. 4:**
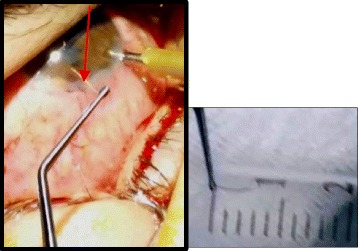
Surgical removal of a 6-mm-long foreign body (*arrow*) via a limbal incision under peri-ocular anesthesia

Further evaluation by AS-OCT (DRI-OCT Triton) demonstrated on the iris surface a highly reflective linear structure with marked posterior shadowing suggesting a foreign body, and associated adjacent moderately reflective round lesion corresponding to the inflammatory exudate seen clinically (Fig. 2). The patient received topical and subconjunctival corticosteroid therapy. As a result, progressively, the iris granuloma decreased in size and the inflammatory exudate resolved, and a foreign body became evident on the iris surface 2 weeks after initiation of treatment (Fig. 3). The IOFB was removed with a toothed grasping intraocular forceps via a single limbal incision (Fig. 4). The macroscopic aspect of the IOFB was an integral vegetable thorn.

**Fig. 5:**
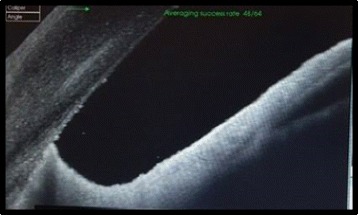
AS-OCT scan of the right eye after removal of the IOFB showed no retained scarps of the IOFB

**Fig. 6:**
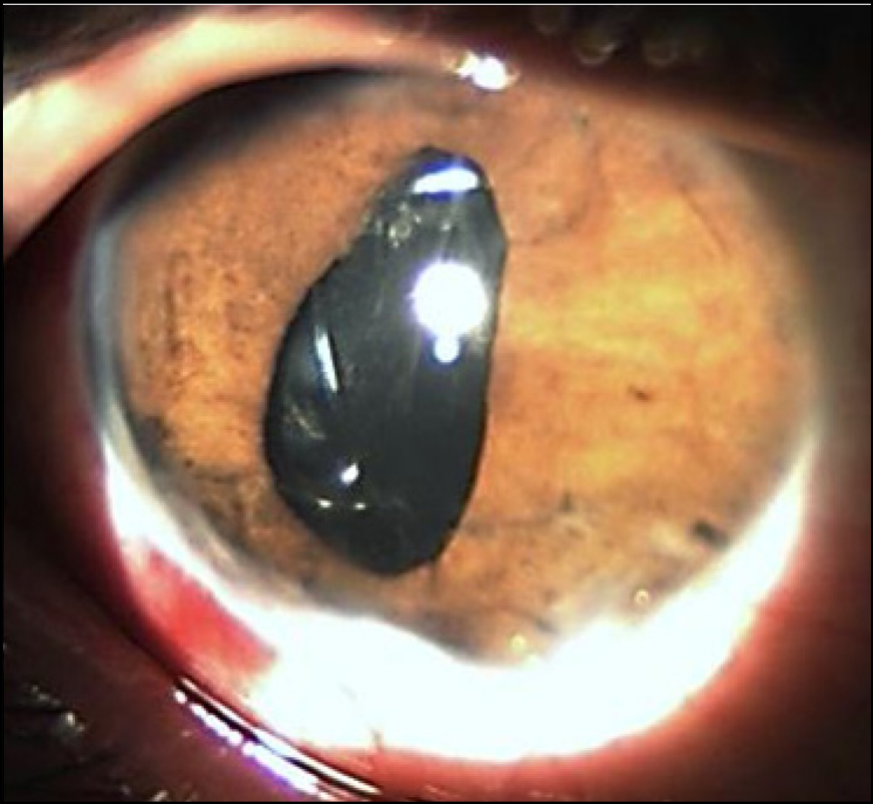
Slit-lamp photograph after successful cataract surgery and lens implantation. The pupil remains irregular

The postoperative period was uneventful and a second AS-OCT showed no IOFB scarps (Fig. 5). Two months later, the patient underwent a successful cataract surgery with intraocular lens implantation. After a 6-month follow-up, visual acuity was 20/40, the anterior chamber was quiet, and there was a pupil distortion (Fig. 6).

### Discussion

IOFB may be responsible for various signs and symptoms according to its size, composition, location, and ballistics [1]. It may masquerade as idiopathic chronic iridocyclitis. Although the diagnosis of such a masquerade syndrome is usually easy when there is a history of ocular trauma [3], it may be challenging especially when the patient denies or forgets the traumatic event [1].

We report here a delayed presentation of a vegetal IOFB located in the anterior segment. The size and the composition of this IOFB and the inflammatory reaction prevented its detection by ophthalmologic examination and the various standard imaging modalities. Only AS-OCT allowed detecting the IOFB.

Previous reports assessed the role of retinal OCT in the detection of small retinal IOFB [4]. AS-OCT enables the visualization of the cornea, iris, ciliary body, and angle. In ocular trauma, AS-OCT results can be used to support diagnosis, to determine the exact location of foreign bodies, to assess the status of surrounding ocular structures, and to monitor the healing process after surgical repair [5]. Many reports underlie its usefulness in intracorneal foreign bodies [5–7].

Reflectivity of IOFB on AS-OCT may vary according to its type [6]. IOFB of glass are well delineated with no internal reflectivity, whereas those of wood exhibit moderate internal reflectivity and those of metal exhibit high anterior reflectivity with shadowing [6].

To the best of our knowledge, our case report is the first to demonstrate the ability of AS-OCT to detect vegetal IOFB that can be missed on clinical examination and by other imaging modalities.

AS-OCT is a safe and non-contact, non-invasive method of anterior ocular tissue imaging [6]. It is superior to other imaging techniques for detecting low-density objects such as wood and vegetable matters, and no contraindication is currently identified [5].

The rationale for using AS-OCT in this case is to reveal unexpected IOFB because neither routine slit-lamp examination nor other imaging techniques were able to demonstrate the presence of such a small IOFB and to visualize how it is positioned in relation to the iris and the ciliary body. Thus, AS-OCT helped us to choose the most appropriate technique for IOFB removal avoiding unexpected intraoperative issues. AS-OCT can be used to prepare high-quality cross-sectional and 3D images of the anterior segment.

In conclusion, IOFB is rather variable in presentation, outcomes, and prognosis. AS-OCT is a valuable imaging modality for detecting and managing vegetal anterior segment IOFB.

## Abbreviations

AS-OCT: Anterior segment optical coherence tomography
IOFB: Intraocular foreign body
LE: Left eye
RE: Right eye
